# Involvement of adiponectin in the pathogenesis of dystrophinopathy

**DOI:** 10.1186/s13395-015-0051-9

**Published:** 2015-08-07

**Authors:** Michel Abou-Samra, Sophie Lecompte, Olivier Schakman, Laurence Noel, Marie C. Many, Philippe Gailly, Sonia M. Brichard

**Affiliations:** Endocrinology, Diabetes and Nutrition Unit, Institute of Experimental and Clinical Research, Medical Sector, Catholic University of Louvain, 1200 Brussels, Belgium; Cellular and Molecular Unit, Institute of Neuroscience, Medical Sector, Catholic University of Louvain, 1200 Brussels, Belgium; Experimental Morphology Unit, Institute of Experimental and Clinical Research, Medical Sector, Catholic University of Louvain, 1200 Brussels, Belgium

**Keywords:** Adiponectin, Mdx, Inflammation, Skeletal muscle, AMPK signaling, NF-κB, Utrophin

## Abstract

**Background:**

The hormone adiponectin (ApN) is decreased in the metabolic syndrome, where it plays a key pathogenic role. ApN also exerts some anti-inflammatory effects on skeletal muscles in mice exposed to acute or chronic inflammation. Here, we investigate whether ApN could be sufficiently potent to counteract a severe degenerative muscle disease, with an inflammatory component such as Duchenne muscular dystrophy (DMD).

**Methods:**

Mdx mice (a DMD model caused by dystrophin mutation) were crossed with mice overexpressing ApN in order to generate mdx-ApN mice; only littermates were used. Different markers of inflammation/oxidative stress and components of signaling pathways were studied. Global force was assessed by in vivo functional tests, and muscle injury with Evans Blue Dye (EBD). Eventually, primary cultures of human myotubes were used.

**Results:**

Circulating ApN was markedly diminished in mdx mice. Replenishment of ApN strikingly reduced muscle inflammation, oxidative stress, and enhanced the expression of myogenic differentiation markers along with that of utrophin A (a dystrophin analog) in mdx-ApN mice. Accordingly, mdx-ApN mice exhibited higher global force and endurance as well as decreased muscle damage as quantified by curtailed extravasation of EBD in myofibers. These beneficial effects of ApN were recapitulated in human myotubes. ApN mediates its protection via the adiponectin receptor 1 (AdipoR1, the main ApN receptor in muscle) and the AMPK-SIRT1-PGC-1α signaling pathway, leading to downregulation of the nuclear factor kappa B (NF-κB) and inflammatory genes, together with upregulation of utrophin.

**Conclusions:**

Adiponectin proves to be an extremely powerful hormone capable of protecting the skeletal muscle against inflammation and injury, thereby offering novel therapeutic perspectives for dystrophinopathies.

**Electronic supplementary material:**

The online version of this article (doi:10.1186/s13395-015-0051-9) contains supplementary material, which is available to authorized users.

## Background

Adiponectin (ApN) is a hormone abundantly secreted by adipocytes; its circulating levels are decreased in obesity and in patients meeting the criteria for the metabolic syndrome. ApN can exhibit insulin-sensitizing, fat-burning, and anti-inflammatory properties as well as modulatory effects on oxidative stress [1], thereby thwarting simultaneously several facets of this syndrome [2].

Adiponectin receptors 1 and 2 (AdipoR1 and AdipoR2) serve as major receptors for ApN by activating the AMP-activated protein kinase (AMPK) and peroxisome proliferator-activated receptor alpha (PPARα) pathways, respectively. AdipoR1 is abundantly expressed in skeletal muscle, whereas AdipoR2 is predominantly expressed in the liver [3]. Skeletal muscle is a major target tissue of ApN. Injection of recombinant ApN to mice or more chronic elevation of ApN in transgenic mice clearly demonstrate the potent insulin-sensitizing properties of the molecule in vivo [4]. ApN also enhances muscle fatty acid consumption [4]. Conversely, muscles of ApN-deficient mice were insulin-resistant [5] and displayed higher susceptibility to oxidative stress, inflammation, and apoptosis; all these abnormalities were exacerbated by acute or chronic inflammatory challenge and corrected by local electrotransfer of the ApN gene [6, 7]. Mice with muscle-specific disruption of AdipoR1 also showed muscle insulin resistance, decreased exercise capacity, mitochondrial content, and oxidative metabolism, as well as enhanced oxidative stress [8]. The presence/action of ApN is therefore important not only to regulate muscle fuel partitioning/metabolism but also to counterbalance inflammation or cellular stress in this organ [6, 7].

Duchenne muscular dystrophy (DMD) is the most frequently inherited human myopathy and the most devastating type of muscular dystrophy. DMD stems from X-linked recessive defects in the gene encoding for dystrophin, a key scaffolding protein of the dystroglycan complex [9, 10]. This complex connects the actin cytoskeleton of myofibers to the extracellular matrix and is thus crucial for maintaining cell membrane stability/permeability as well as normal contractile function of the skeletal muscle. Disruption of this complex leads to membrane damage allowing massive infiltration of immune cells, chronic inflammation, necrosis, and severe muscle degeneration [9]. Normally, muscle cells possess the capacity to regenerate in response to injury signals. However, this ability is progressively lost in DMD, due to an exhaustion of satellite cells during ongoing degeneration/regeneration cycles [10]. Although dystrophin mutations represent the primary cause of DMD, it is the secondary processes involving persistent inflammation and subsequent impaired regeneration that likely exacerbate disease progression [11].

The aim of this work was to explore whether ApN may play a beneficial role in DMD. To this end, dystrophin-deficient mdx mice, a widely used mouse model of DMD, were crossed with our transgenic mice, which moderately overexpress ApN [12]. These transgenic mice with homotopic overexpression of native ApN showed a moderate elevation of circulating ApN with no change in the distribution of ApN multimers [12]. We thus examined whether the resulting mdx-ApN mice exhibited a lower degree of muscle inflammation, oxidative stress, and injury together with improved muscular function. Lastly, we unraveled the mechanisms underlying the protection afforded by ApN.

## Methods

### Animals

Transgenic C57BL/6J mice overexpressing adiponectin (ApN mice), which had been generated in our lab, were used; native full-length ApN was placed under the control of the adipocyte aP2 promoter, thereby targeting ApN expression to white adipose tissue. As ApN is secreted, these mice also showed a moderate elevation of circulating ApN levels [12]. C57BL/10ScSn mdx mice were purchased from Jackson Laboratory (Maine, USA) and crossed with ApN mice in order to obtain mdx mice overexpressing ApN (mdx-ApN mice). Three groups of F_1_ mice were studied in all experiments: the mdx-ApN mice, their mdx littermates (true controls), and wild-type (WT) mice; the three groups of mice were maintained on the same C57BL/6J-10 ScSn genetic background. Only male mice were studied.

These animals were maintained under a standard laboratory chow and housed at a constant temperature with a fixed light-dark cycle. At the end of the experiments, mice were sacrificed by cervical dislocation (between 09.00 and 11.00 h). Blood samples were saved. Pairs of *tibialis anterior* (TA) muscles or inguinal fat pads were weighed, frozen in liquid nitrogen, and stored at −80 °C for subsequent analyses. For some experiments dealing with evaluation of muscle injury, additional muscles were sampled.

All procedures were approved by the Ethical Committee for Animal Experimentation from the Medical Sector at the Catholic University of Louvain.

### Light microscopy, immunohistochemistry, and morphometry

Muscle samples were fixed in 10 % formalin for 24 h and embedded in paraffin. Sections 5 μm thick were stained with hematoxylin-erythrosin-safran (HES). The percentage of muscle fibers with central nuclei (a characteristic feature of DMD) was determined on more than 1000 fibers per muscle section. For immunohistochemistry, sections were processed as previously described [6] using rabbit polyclonal antibodies directed against peroxiredoxin 3 (PRDX3, dilution 1:700, incubation 2 h), peroxiredoxin 5 (PRDX5, 1:300, 2 h) [gifts from B. Knoops, University of Louvain, Brussels, Belgium [13]], 4-hydroxy-2-nonenal (HNE, 1:100, 2 h), tumor necrosis factor alpha (TNFα, 1:200, 2 h), interleukin-1β (IL-1β, 1:300, overnight), and nuclear factor kappa B (NF-κB, 1:50, overnight) (all from Abcam, Cambridge, UK). Rat monoclonal antibodies directed against markers of macrophages (CD68) or of T lymphocytes (CD3) (1:50, overnight, Abcam) were also used. Before immunostaining, sections were submitted to heat-mediated antigen retrieval using a microwave oven and Tris-citrate buffer (pH 6.5). Binding of antibodies was detected by applying for 30 min at room temperature a second antibody, which was a biotinylated goat anti-rabbit IgG (H+L) or a biotinylated rabbit anti-rat IgG (H+L) (Labconsult, Brussels, Belgium). Peroxidase activity was revealed with 3,3′-diaminobenzidine (DAB) (Life Technologies, Gent, Belgium), which produces a brown staining. For each marker, all slides from the three groups of mice were treated simultaneously for immunohistochemistry analysis and DAB revelation, and then analyzed together. Immunohistochemical controls were performed by omission of the first antibody or of the first and second antibodies or by using pre-immune serum. For quantification of NF-κB expression, the percentage of immunolabeled nuclei was determined after counting an average of 1000 fiber nuclei per slide for each mouse. For PRDX3/5, HNE, TNFα, IL-1β, CD3, and CD68, whole muscle section were scanned using the Leica SCN400 slide scanner (Leica Microsystems, Diegem, Belgium), and then the percentage of DAB surface area within muscle fibers was quantified using the Tissue Image Analysis 2.0 (Leica).

### In vivo studies of global force or resistance

Mice were submitted to three main tests:

- *Wire test.* Animals were suspended by their forelimbs from a 1.5 mm-thick, 60 cm-long metallic wire at 45 cm above soft ground. The time (seconds) until the mouse completely released its grasp and fell down was recorded. Three trials were performed per session, with a 30-s recovery period between trials. The maximum time per trial was set to 180 s. For each mouse, the scores of the three trials were averaged [14].
- *Grip test.* The grip strength test measures the muscle strength of forelimb or of combined fore- and hindlimb muscles. Limb strength was recorded using a grid connected to a sensor (Panlab-Bioseb, Vitrolles, France). The mice were gently laid on the top of the grid so that their front paws (forelimb test) or both fore and hind paws (combined test) can grip the grid. Then, mice were pulled back steadily until the grip was released down the complete length of the grid. Each test was repeated three times at an interval of 20 min. Results are presented as the mean of the two highest values of force recorded, related to body weight [15].
- A third test of *eccentric exercise* was also performed mainly to evaluate muscle injury (see below).

### Quantification of muscle damage markers in plasma

Plasma creatine kinase (CK) and lactate dehydrogenase (LDH) activities were quantified to evaluate skeletal muscle damage as injured muscles release CK and LDH into the bloodstream at high levels. Kits were based on colorimetric methods (Gentaur, Kampenhout, Belgium). CK and LDH activities were expressed as IU/L.

### Evaluation of muscle injury after in vivo staining with Evans blue dye

*Evans blue dye* (EBD) irreversibly binds to plasma albumin in vivo, and upon injury, the complex leaks into the surrounding tissues [16, 17]. EBD (Sigma-Aldrich, Bornem, Belgium) was dissolved in PBS buffer, sterilized by filtration through membrane with pore size of 0.2 μm, and kept at 4 °C. The dye solution was injected *ip* into the mice (1 mg dye/0.1 ml/10 g body weight). The mice were sacrificed 24 h later either in basal (sedentary) conditions or after bouts of eccentric exercise [18].

*Eccentric exercise*. The mice were subjected to a downhill running exercise on a treadmill with a downward inclination of 15°, and at a speed of 10 m/min for 10 min. This training was repeated daily for 3 days, and the mice were sacrificed 1 h after the 3rd session and 24 h after the EBD injection [18]. Six different muscles were studied: *biceps brachii* (BB) and *triceps brachii* (TB) from the forelimbs; TA, *gastrocnemius* (G), *extensor digitorium longus* (EDL), and *soleus* (S) from the hindlimbs.

*Spectrophotometric detection of EBD*. EBD was extracted from powdered muscle using dimethylformamide, and the absorbance was read spectrophotometrically at 620 nm. The extravasated EBD concentration was calculated against a standard curve and was expressed as ng of EBD/mg muscle weight [16].

*Fluorescence microscopy detection of EBD*. Muscles were embedded in Tissue-Tek (Sakura, Alphen-aan-den-Rijn, Netherlands) using iso-pentane frozen in liquid nitrogen. Three different transverse cryostat sections (10 μm thick) were obtained for each muscle and observed by fluorescence microscopy [17].

### Western blotting and immunoprecipitation

Skeletal muscle was homogenized in a lysis buffer (Cell Signaling Technology, BIOKE, Leiden, The Netherlands) supplemented with 100 mM NaF and 1 % protease inhibitor cocktail (Active Motif, Rixensart, Belgium). Immunoblotting was performed as reported [19] by using rabbit polyclonal antibodies directed against phospho-NF-κB p65 (phosphor Ser536) and myogenic factor 5 (Myf5) (both from Abcam); AMPK, P-AMPK, and Sirtuin 1 (SIRT1) (all form Cell Signaling); peroxisome proliferator-activated receptor-γ coactivator-1α (PGC-1α) and Utrophin (both from Santa Cruz, Heidelberg, Germany); or a mouse monoclonal antibody directed against myogenic differentiation (MyoD) (Abcam). Signals were revealed by enhanced chemiluminescence, then quantified and normalized to those of actin, Ponceau stain or the studied protein without post-translational modifications (when these modifications were studied) using ImageJ program (National Institutes of Health, Maryland, USA).

To obtain protein extracts for immunoprecipitation, skeletal muscle was homogenized in the lysis buffer indicated above, supplemented with 1 % protease/phosphatase inhibitor cocktail (Cell Signaling), 5 mM nicotinamide, and 1 mM sodium butyrate. A total of 1000 mg of proteins from muscle samples were used. The immunoprecipitation was carried out using magnetic beads (Thermo Fisher Scientific, Lafayette, CO) and anti-PGC-1α antibody (2 μl antibody/1 g total protein; Santa Cruz) following the manufacturer’s instructions. The beads were collected using neodymium magnets and washed with TBS-Tween 0.05 %. After the final wash, the samples were dissolved in 100 μl of elution solution (0.1 M glycine, pH 2.5); 20 μl were then separated by SDS-PAGE and immunoblotted, as described [20], using an acetyl-lysine antibody (1:1.000; Cell Signaling) and anti-PGC-1α antibody (1:1.000; Santa Cruz), respectively.

### Culture of human myotubes

Primary cultures of human skeletal muscle cells were initiated from satellite cells of quadriceps samples obtained from male organ donors (*n* = 5, 40 ± 9 years) or from *latissimus dorsi* flap samples obtained from women undergoing reconstructive surgery after breast cancer (*n* = 6, 48 ± 13 years). The last series of biopsy samples were used for siRNA transfection experiments. For each patient, the culture experiments were done in duplicate and the data from a given individual were then averaged. Patients provided written informed consent, and the study protocol had the approval of the local Ethical Committee of Saint-Luc University Hospital.

Cultures were performed as described [21] with minor modifications. Myoblasts were grown in 35-mm (or 6 wells; siRNA transfection) plates at 37 °C in the presence of 5 % CO_2_ in F-12 (Ham) supplemented with 12 % fetal bovin serum (FBS), 1 % l-glutamine, and 100 μg/ml Primocin™ (Invivogen, Toulouse, France) (all other products from Life Technologies, Inc., Merelbeke, Belgium). After 3–5 days of proliferation, at the end of which the seeding density has reached 70–80 %, the growth medium was replaced by the fusion medium which consists of 1 part DMEM, 1 part F-12 (Ham), 2 % horse serum (HS), 1 % l-glutamine, and 1 % penicillin-streptomycin (Life Technologies). This fusion medium was then changed every 2 days, and differentiation was allowed to continue for 10 days (time required to obtain mature myotubes with characteristic elongated and multinucleated morphology) before the experimentation period.

Ten days after differentiation, cells were treated with human recombinant TNFα (10 ng/ml) + interferon gamma (IFNγ) (10 ng/ml) and/or ApN (5 μg/ml), at the indicated concentrations, for 24 h (TNFα from Tebu-Bio, Boechout, Belgium, IFNγ from R&D systems, Abington, UK, and ApN from Biovendor, Heidelberg, Germany). In some experiments, cells were first transfected before inflammatory challenge and/or ApN treatment. Briefly, 3.10^5^ cells/well were transfected with either the On-Targetplus non-targeting pool siRNAs (negative control, NT siRNAs), or a specific On-Targetplus siRNA SMARTpool against human AdipoR1 (50 nM) or human SIRT1 (50 nM) or human PGC-1α (70 nM) (all from Dharmacon, Thermo Fisher Scientific) using 7 μl Lipofectamine RNAiMAX reagent (Life Technologies) for 24 h. siRNA silencing was effective, ranging from 70 to 95 % in all experiments. Next, the medium was renewed and cells were treated with TNFα + IFNγ with or without ApN for an additional 24 h. At the end of the experiments, cells were collected and rinsed twice in PBS before RNA extraction.

### RNA extraction and real-time quantitative PCR (RT-qPCR)

RNA was isolated from cultured cells and from mouse adipose tissue with TriPure reagent (Roche Diagnostics, Vilvoorde, Belgium). Of the total RNA, 1–2 μg were reverse transcribed, as described previously [21]. RT-qPCR primers were designed for mouse cyclophilin, ApN as reported [21], for interleukin 10 (IL-10; sense, 5′-TGA TGC CCC AGG CAG AGA AGC A-3′; antisense, 3′-GGG GAG AAA TCG ATG ACA GCG CCT C-5′), myosin heavy chain 7 (Myh7; sense, 5′-GGT GCC AAG GGC CTG AAT GAG GAG-3′; antisense, 3′-GGT CTG AGG GCT TCA CGG GCA C-5′), myosin heavy chain 1 (Myh1; sense, 5′-AGC TTC AAG TTT GGA CCC ACG GTC G-3′; antisense, 3′-GCA GCC TCC CCG AAA ACG GC-5′), myosin heavy chain 6 (Myh6/Mrf4; sense, 5′-TGC GGA TTT CCT GCG CAC CT-3′; antisense, 3′-GCA TCC ACG TTT GCT CCT CCT TCC-5′), myosin heavy chain 3 (Myh3/eMyHC; sense, 5′-CAG AAA TGG AGA CAC GGA TCA GA-3′; antisense, 3′-AGA GGT GAA GTC ACG GGT CTT TGC C-5′), myogenin (MyoG; sense, 5′-CCA GCC CAT GGT GCC CAG TGA A-3′; antisense, 3′-TGT AGG GTC AGC CGC GAG CAA-5′), and utrophin A (sense, 5′-GCC AGA CAC TAT GAC CCC TCC CA-3′; antisense, 3′-GGG CAT GCT CAT CCT CCA CGC T-5′). RT-qPCR primers for human TATA box-binding protein (TBP), TNFα, interleukin-6 (IL-6) were similar to those previously reported [22], for IκB (sense, 5′-TGT GGC AAC TGG AAG CAA GCC C-3′; antisense, 3′-ACC ATG GCC GCA TCA ATG TGC T-5′), and utrophin A (sense, 5′-AGT TCC ACC AGG CAG CGG GAG-3′; antisense, 3′-ACC TCC GTG AGA TCC GTG CTG G-5′). Cyclophilin (mouse) and TBP (human) were used as reporter genes. Of the total RNA equivalents, 40–80 ng were amplified using an iCycler iQ real-time PCR detection system (Bio-Rad Laboratories, Nazareth, Belgium) [21]. The threshold cycles (Ct) were measured in separate tubes and in duplicate. The identity and purity of the amplified product were checked by electrophoresis on agarose minigels, and analysis of the melting curve was carried out at the end of the amplification. To ensure the quality of the measurements, each plate included a negative control for each set of primers.

### Result presentation and statistical analysis

Results are means ± SD for the indicated number of mice or experiments. Comparisons between the three groups of mice (WT, mdx, and mdx-ApN) were carried out by one-way ANOVA followed by Tukey’s test (Prism 5; GraphPad Software, California, USA)*.* When more than three experimental conditions were compared in vitro, the influence of ApN and that of TNFα/IFNγ were assessed by two-way ANOVA with F test, followed by *post hoc* two by two comparisons with Bonferroni correction for multiple comparisons (Prism 5). Comparisons between two myotubes conditions from a given subject were made using two-tailed paired Student’s *t* test. Differences were considered statistically significant at *P* < *0.05.*

## Results

### Characteristics of mdx mice overexpressing adiponectin

Because skeletal muscle of mdx mice exhibit degenerative-regenerative cycles between 2 and 14 weeks of age, we chose mid-stage mice for most experiments [23]. We thus compared three groups of 8-week-old mice: the mdx mice overexpressing ApN, their mdx littermates (true controls), and WT mice (Table 1).

**Table 1:** Characteristics of mdx mice overexpressing adiponectin

	WT	mdx	mdx-ApN
Body weight (g)	22.2 ± 0.6	23.1 ± 0.7	22.2 ± 0.9
Tibialis anterior muscle pair weight (mg)	72 ± 2	92 ± 6**	76 ± 5***
Plasma ApN (μg/ml)	4.2 ± 0.2	2.6 ± 0.2**	3.6 ± 0.1*^/^***
Adipose tissue ApN (mRNA levels)	1.0 ± 0.2	0.5 ± 0.1**	1.4 ± 0.2*^/^***

Three groups of mice were compared at the age of 8 wks: the mdx-ApN mice (mdx mice overexpressing ApN), their mdx littermates (true controls), and wild-type (WT) mice. Blood, *tibialis anterior* muscle and inguinal adipose tissue were sampled between 09.00 and 11.00 h. ApN was measured in plasma by ELISA, and in adipose tissue by RT-qPCR. In this case, ApN gene expression was normalized to the levels of cyclophilin and presented as relative expression compared to WT values. Data are means ± SD for six mice per group

*p < 0.01; **p < 0.001 compared to WT; ***p < 0.001 compared to mdx

There were no significant differences in body weight between any groups. However, as expected [24], mdx mice presented an increase in *tibialis anterior* mass, which was corrected in mdx-ApN mice (Table 1).

Remarkably, circulating ApN was decreased by ~40 % in mdx mice compared to WT mice. This decrease was explained by halved expression of ApN in fat tissue, the major contributor to systemic levels. Compared to mdx controls, plasma ApN was increased in mdx-ApN mice (and was almost completely normalized), in line with the 3-fold rise of ApN mRNAs in adipose tissue, where the transgene under the control of the adipocyte aP2 promoter is overexpressed (Table 1) [12].

### Effects of adiponectin on markers of inflammation and oxidative stress in mdx muscles

We tested the hypothesis that ApN replenishment could slow down the progression of dystrophic pathology by counteracting excessive inflammation/oxidative reactions.

**Fig. 1:**
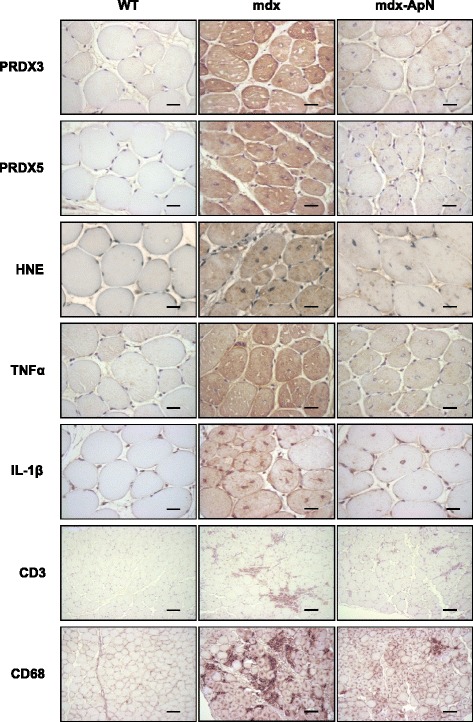
Effects of adiponectin on different markers of oxidative stress and inflammation in muscles of mdx mice. *Tibialis anterior* muscles were sampled from 8-week-old WT mice, mdx mice, or mdx mice overexpressing adiponectin (mdx-ApN). Immunodetection was performed with specific antibodies directed against three oxidative stress markers (PRDX3/5, HNE), two pro-inflammatory cytokines (TNFα, IL-1β), one marker of T lymphocytes (CD3), and one macrophage marker (CD68). Representative sections for six mice per group are shown. Scale bar = 25 μm (for PRDX3/5, HNE, TNFα, and IL-1β) and 100 μm (for CD3 and CD68)

**Fig. 2:**
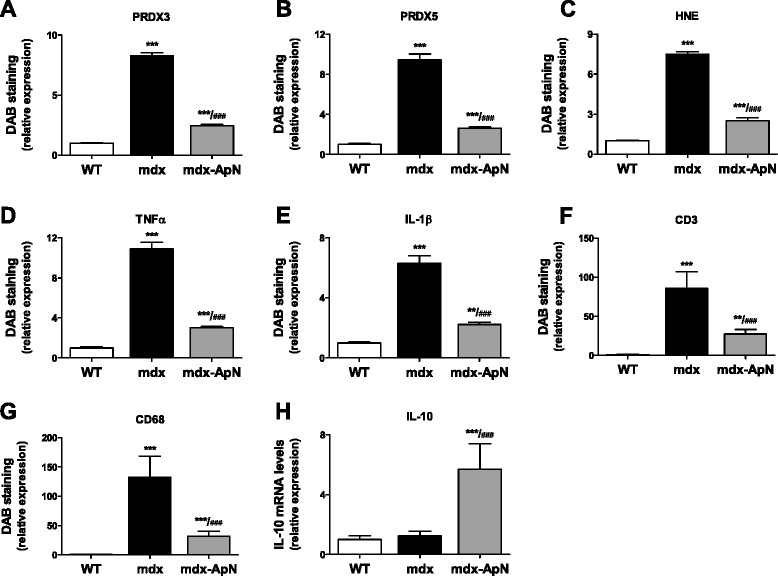
Quantification of markers of oxidative stress and inflammation in 8-week-old mdx mice. For each immunolabeling of Fig. 1 (**a–g**), the percentage of DAB deposit areas was calculated within muscle. **h** mRNA levels of IL-10, an anti-inflammatory cytokine. mRNA levels were normalized to cyclophilin. Data are means ± SD for six mice per group and the subsequent ratios were presented as relative expression compared to WT values. **p < 0.01; ***p < 0.001 vs. WT; ###*p* < 0.001 vs. mdx

**Fig. 3:**
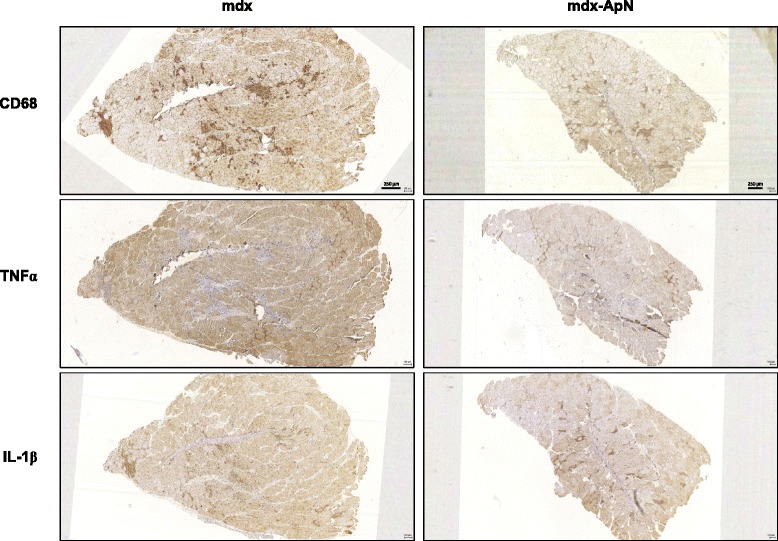
Inflammation in the skeletal muscle of mdx mice. Successive sections of *Tibialis anterior* muscles from 8-week-old mdx and mdx-ApN mice were stained for CD68, TNFα, and IL-1β. Representative sections for six mice per group are shown. Scale bar = 250 μm

When compared to WT mice, myofibers from *tibialis anterior* of mdx mice displayed a strong immunolabeling for PRDX3/5, two markers of oxidative stress, and for HNE, a lipid peroxidation product (Fig. 1). Quantification of this immunolabeling showed that the extent of DAB staining within myofibers was ~7.5- to 9.5-fold higher in mdx than in WT mice (see Fig. 2a–c). Qualitatively similar results were observed for TNFα and for IL-1β, two pro-inflammatory cytokines (Fig. 1), with an extent of DAB staining being 11- and 6-fold greater in mdx than in WT mice, respectively (Fig. 2d, e). In addition, a massive infiltration of both T lymphocytes (immunolabeling for CD3) and macrophages (CD68, a marker of the pro-inflammatory M1 phenotype; [25]) was observed in mdx mice, but not in WT mice (Fig. 1 and Fig. 2f, g). All these indicators of oxidative stress and inflammation were remarkably reduced in mdx-ApN mice (Figs. 1 and 2). Serial muscle cross-sections revealed that both myocytes and macrophages contribute to the production of pro-inflammatory cytokines. In mdx mice, areas that were positive for C68 were negative for TNFα, while this cytokine was abundant within the myocytes. A reverse pattern of expression was however observed for IL-1β, which was mainly produced by macrophages and less by myocytes (Fig. 3). Remarkably, gene expression of IL-10, an anti-inflammatory cytokine that activates the M2 macrophage phenotype [25] was also markedly increased in mdx-ApN mice (Fig. 2h). Taken together, these data suggest that ApN is able to strongly defend the skeletal muscle against excessive inflammatory reactions and oxidative stress.

This effect of ApN persisted later in life. In 1-year-old mice, oxidative stress and inflammation were still blunted in mdx-ApN muscles, as shown by reduced expression of HNE and TNFα (Additional file 1: Figure S1).

### Effects of adiponectin on markers of muscle differentiation in mdx mice

**Fig. 4:**
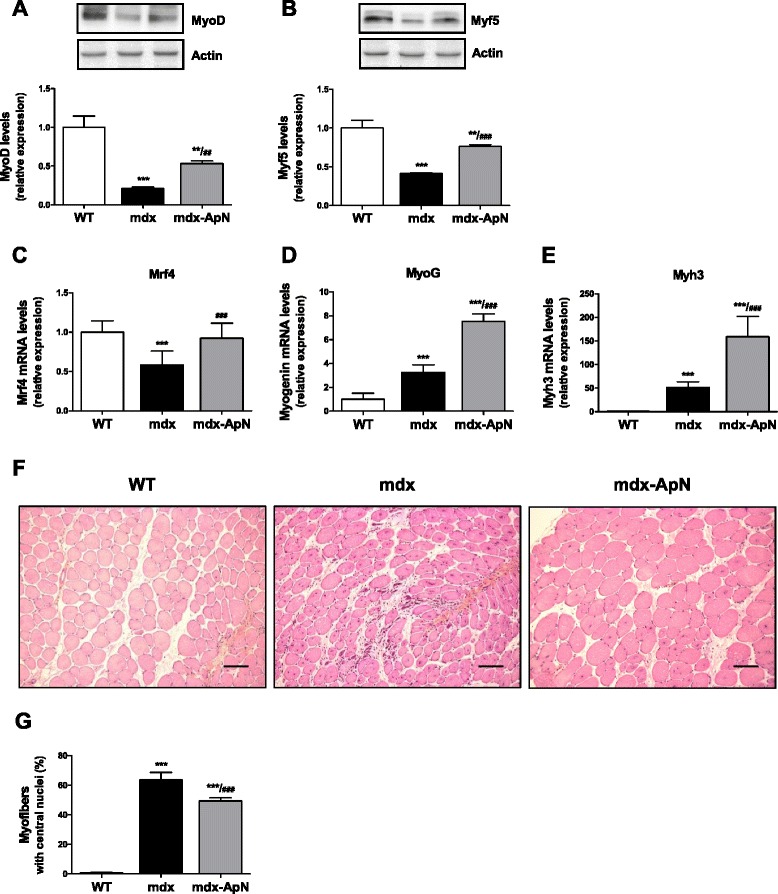
Effects of adiponectin on markers of the myogenic program in mdx mice. The expression of MyoD (**a**) and Myf5 (**b**), two myogenic regulatory factors, was analyzed by Western blotting in *tibialis anterior* muscles from the three groups of mice. Levels of each marker were normalized to actin levels. mRNA levels of Mrf4 (**c**) and myogenin (**d**), two markers of muscle differentiation. **e** mRNA levels of Myh3 (eMyHC), a marker of skeletal muscle regeneration. mRNA levels were normalized to cyclophilin and the subsequent ratios were presented as relative expression compared with WT values. **f** *Tibialis anterior* sections stained with hematoxylin-erythrosin-safran. Scale bar = 100 μm. **g** The percentage of muscle fibers with central nuclei was counted. Results are means ± SD; *n* = 6 mice per group. **p < 0.01; ***p < 0.001 vs. WT; ##*p* < 0.01; ###*p* < 0.001 vs. mdx mice

Since ApN has been found to induce myogenesis in C2C12 cells [26], we explored different key markers involved in the skeletal myogenic program [25]. The expression of MyoD and Myf5 proteins, that activate satellite cells to proliferate [25], was markedly reduced in *tibialis anterior* of mdx mice when compared to WT mice (-80 and −60 %, respectively). The decrease of these proliferation markers, which may be ascribed to the inflammatory context [27, 28], was partially restored in mdx-ApN mice (Fig. 4a, b). Two later markers (early differentiation phase), myogenin and Mrf4, were next studied. Mrf4 showed a pattern of expression similar to that of the proliferation markers, while myogenin was increased in mdx mice and further upregulated in mdx-ApN ones (Fig. 4c, d). The regeneration marker, the embryonic myosin heavy chain (Myh3) behaved similarly (Fig. 4e).

The proportion of fibers with central nuclei, a hallmark of dystrophic muscle, was almost undetectable in WT mice (<1 %), amounted to 64 % in mdx mice, and was reduced to 49 % in mdx-ApN mice (Fig. 4f, g). This reduction suggests improved myogenic program and more complete muscle regeneration.

### Effects of adiponectin on global force and resistance of mdx mice

**Fig. 5:**
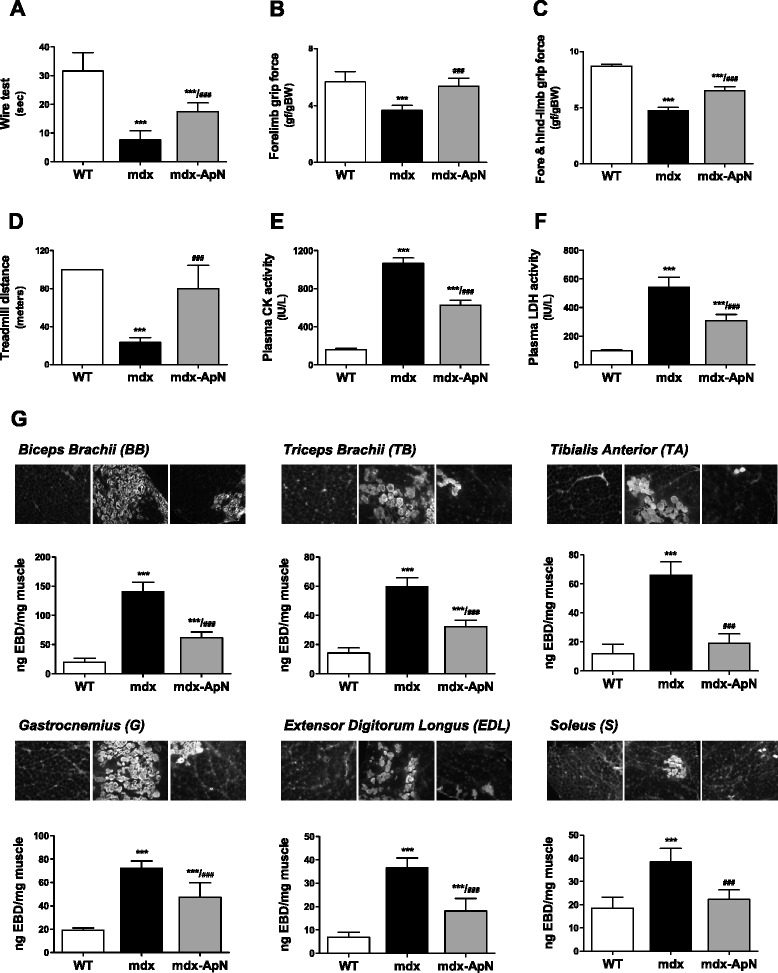
Effects of adiponectin on global force, resistance and muscle injury in mdx mice. Functional in vivo studies were carried out in mice from the three groups. **a** The animals were subjected to a wire test where they were suspended by their forelimbs and the time until they completely released the wire and fell down was recorded (seconds). **b**, **c** The mice were also lowered on a grid connected to a sensor to measure the muscle force of their forelimbs (**b**) or of both fore- and hindlimbs (**c**); data were then expressed in gram-force relative to body weight (gf/g BW). **d** Mice were submitted to a downhill treadmill exercise for 10 min during three consecutive days. On the 3rd session, the covered distance (meters) were measured for each mouse, 100 m being the maximal distance. Muscle injury was assessed by plasma activity of CK and LDH expressed as IU/L (basal state) (**e**, **f**) and by using EBD (**g**). Quantification of EBD extravasation was measured in different muscles after exercise. Six different muscles were sampled: *biceps brachii*, *triceps brachii*, *gastrocnemius*, *tibialis anterior*, *extensor digitorium longus*, and *soleus*. Qualitative detection of EBD was evaluated by fluorescence microscopy on frozen cryostat sections (see *insets* above histograms). Extravasated EBD concentrations were also quantified spectrophotometrically after extraction of the dye. Data were expressed as ng of EBD/mg muscle weight. The results presented herein are the means ± SD; *n* = 9 (**a–c**) and six (**d–g**) mice per group. ***p < 0.001 vs. WT ; ###*p* < 0.001 vs. mdx mice

We used two different functional tests to investigate in vivo the global muscular force: the wire test and the grip test. The wire test evaluates muscle force and resistance to fatigue: the mouse is suspended from a horizontal wire and the time until the mouse releases its grasp is recorded [14]. Mdx mice fell down much quicker than WT mice, while mdx-ApN mice showed intermediate resistance (Fig. 5a). The grip test measures strength of forelimb or of combined fore- and hindlimb muscles [15]. The force developed by forelimbs of mdx mice was ~36 % lower than that of WT mice, while ApN overexpression completely rescued forelimb strength (Fig. 5b). When combined fore- and hindlimb force was measured, muscular strength of mdx mice was halved when compared to WT mice, while mdx-ApN mice showed intermediate values (Fig. 5c), indicating enhanced muscle force in the presence of ApN.

Resistance to fatigue was evaluated by an eccentric exercise also designed to investigate muscle injury. On the 3rd day of the exercise, WT mice covered the maximum distance (100 m), the running distance of mdx mice fell drastically (~24 m), while that of mdx-ApN mice was not statistically different from that of WT ones (Fig. 5d and Additional file 2: Movie S2).

### Effects of adiponectin on skeletal muscle injury

Creatine kinase and lactate dehydrogenase activities, two markers of muscle damage in plasma, measured herein in sedentary conditions, were ~6.7- and 5.5-fold higher in mdx than in WT mice, respectively, while both activities declined by over 40 % in mdx-ApN mice (Fig. 5e, f).

Sarcolemmal integrity was more directly evaluated by using EBD. EBD extravasation was quantified in different muscle types either in basal state (not shown) or after repeated downhill treadmill exercise (Fig. 5g). The EBD content of forelimbs was markedly increased in mdx mice (7-fold for BB and 3-fold for TB*)* compared to WT ones, and this content was halved in mdx-ApN mice. Likewise, the EBD content of the four hindlimb muscles tested was 2- to 6-fold higher in mdx mice than in WT ones, and this increase was partially (G, EDL) or completely (TA*,* S) reversed by ApN overexpression (Fig. 5g). The insets illustrate representative muscle sections under fluorescence microscopy: the proportion of damaged (fluorescent) fibers within each muscle corroborates the quantitative data shown in the histograms (Fig. 5g). Hence, ApN partially or totally restored sarcolemmal integrity and abrogated muscle injury in each type of skeletal muscles tested.

### Effects of adiponectin on AMPK signaling pathway

Because ApN activates AMPK by binding to AdipoR1 in muscle, thereby triggering beneficial effects on the metabolic syndrome [8] and because AMPK is involved in skeletal muscle remodeling [29], we explored the AMPK signaling pathway.

**Fig. 6:**
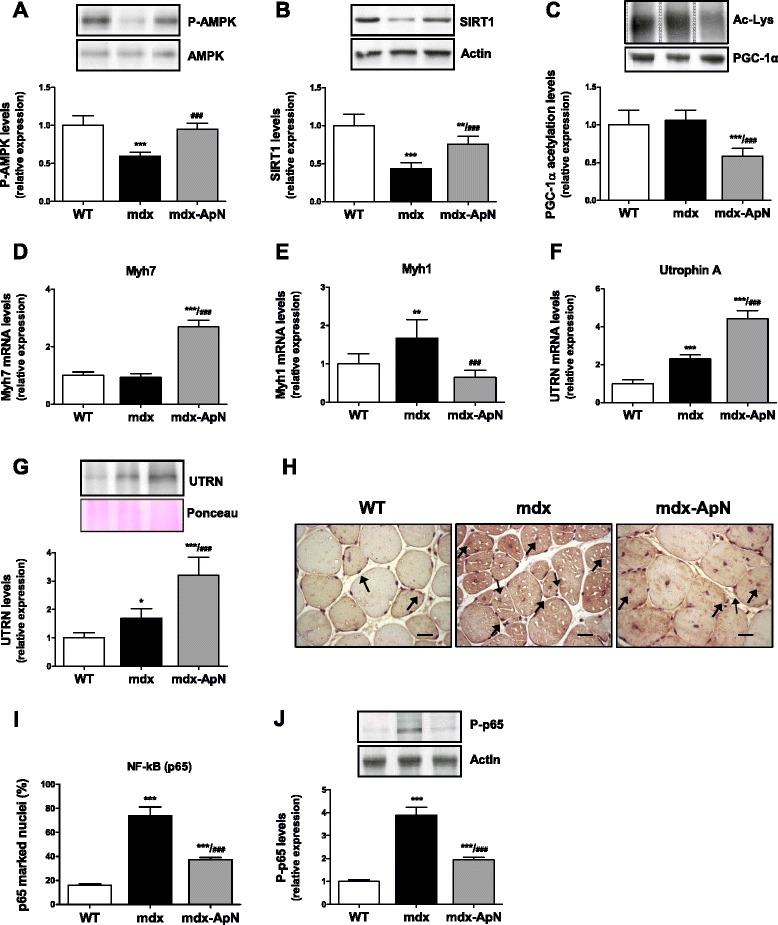
Effects of adiponectin on AMPK signaling pathway and NF-κB activity in *tibialis anterior* muscles of mdx mice. The expression of P-AMPK (phosphorylated form) (**a**) and SIRT1 (**b**) was analyzed by Western blotting in muscles from the three groups of mice. **c** Densitometry of immunoprecipitation experiments performed on skeletal muscle lysates, using anti-PGC-1α antibody for precipitation and anti-acetyl-lysine antibody for immunoblotting. **d** mRNA levels of Myh7, a marker of slow twitch, oxidative myofibers. **e** mRNA levels of Myh1, a marker of fast twitch, glycolytic myofibers. **f** mRNA and **g** protein levels of utrophin A (UTRN) with a representative Western blot and Ponceau S stain. **h** Immunodetection of NF-κB (p65) in *tibialis anterior* sections; some positive marked nuclei (*brown* color) are indicated by *arrows*. Scale bar = 25 μm. **i** Quantification of p65 immunolabeling in myofiber nuclei (expressed as percent of total nuclei) in sections (as those shown in **g**). **j** Immunoblotting of phosphorylated NF-κB p65 in the same muscles. Levels of P-AMPK, SIRT1, and Acetyl-Lys were normalized to AMPK, actin, and PGC-1α levels, respectively. mRNA levels were normalized to cyclophilin, utrophin A protein levels to Ponceau, and P-p65 to Actin. The subsequent ratios were presented as relative expression compared to WT values. Results are means ± SD; *n* = 6 mice per group. *p < 0.05; **p < 0.01; ***p < 0.001 vs. WT; ###*p* < 0.001 vs. mdx mice

The expression of total AMPK was similar in the three groups of mice; however, the active phosphorylated form was lower in the *tibialis anterior* muscle of mdx mice than in WT ones (Fig. 6a). Similarly, the expression of the deacetylase SIRT1 was markedly reduced in mdx mice compared to WT mice (Fig. 6b). Both AMPK phosphorylation and SIRT1 expression were completely or partially restored in the skeletal muscle of mdx-ApN mice (Fig. 6a, b). PGC-1α is a downstream event of this signaling cascade. Expression of total PGC-1α was not modified in any groups and the deacetylated form, which is necessary for PGC-1α activation, was not modified in mdx mice. However, mdx-ApN mice showed a sharp (~40 %) decrease in PGC-1α acetylation (Fig. 6c). Because PGC-1α is a transcriptional coactivator, it has the potential to alter the expression of numerous genes. More specifically, it can induce a myofiber shift toward a more oxidative phenotype with increased expression of utrophin A [30, 31]. Accordingly, expression of Myh7, a specific marker of slow twitch, oxidative (type I) myofibers was increased in *tibialis anterior* of mdx-ApN mice, while that of Myh1, a marker of fast twitch, glycolytic fibers was decreased (Fig. 6d, e). Moreover, both mRNA and protein levels of utrophin A, an autosomal analog of dystrophin [32], which were slightly augmented in regular mdx mice likely to compensate the lack of dystrophin [31], were further upregulated in mdx-ApN mice (~3–4-fold vs. WT) (Fig. 6f, g). Taken together, these data indicate a significant activation of the AMPK-SIRT1-PGC-1α axis in mdx-ApN mice.

### Effects of adiponectin on NF-κB activity in mdx muscles

NF-κB is a pleiotropic transcription factor modulating immune, inflammatory, survival, and proliferative responses [11]. To further characterize the molecular mechanisms responsible for the protective effects of ApN, we examined NF-κB activity by two different approaches.

First, we measured the nuclear translocation of NF-κB p65 subunit. *Tibialis anterior* sections were immunolabeled and the percentage of NF-κB marked nuclei in myofibers was counted: this percentage was 4.5-fold higher in mdx than in WT mice, but this rise was halved in mdx-ApN mice (Fig. 6h, i). Second, we quantified the active phosphorylated form of NF-κB (P-p65 on Ser536) by Western blot (Fig. 6j). P-p65 activation was ~4-fold higher in *tibialis anterior* of mdx mice than in WT mice, and this enhanced activation was again halved in mdx-ApN mice. Hence, the hyperactivity of NF-κB in muscles of mdx mice was attenuated by ApN. This effect persisted in old mice (Additional file 1: Figure S1D).

### Effects of adiponectin on inflamed human myotubes

**Fig. 7:**
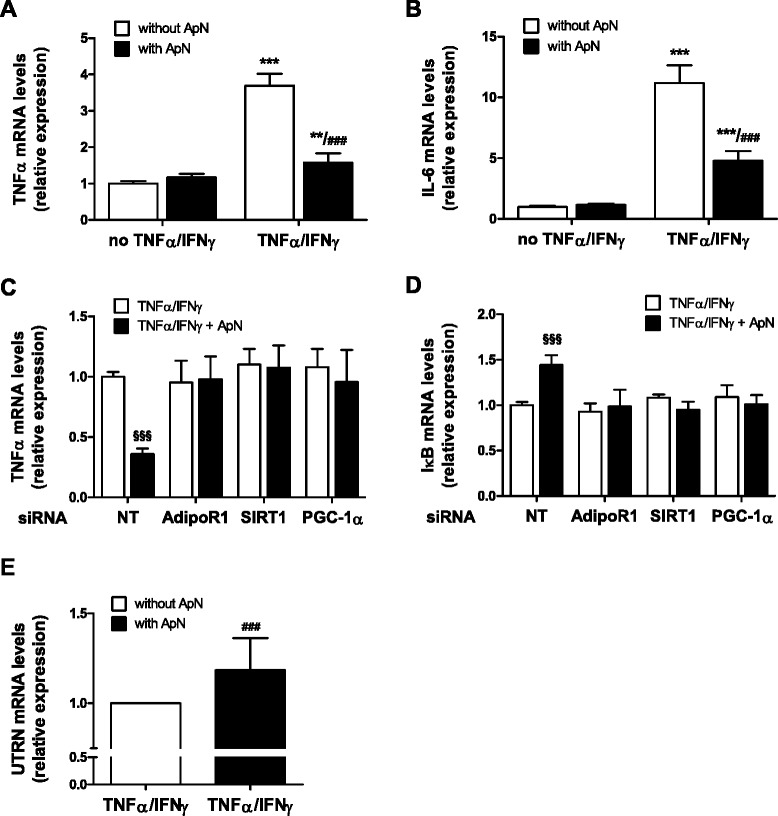
Effects of adiponectin on inflammatory markers in human skeletal muscle. TNFα (**a**) and IL-6 (**b**) mRNAs in primary culture of human myotubes: cells were treated with or without ApN, while being or not challenged with TNFα and IFNγ. TNFα (**c**) and IκB (**d**) mRNAs in human myotubes, which were transfected with siRNAs against AdipoR1, SIRT1, PGC-1α, or a negative (non-targeting, NT) control. After transfection, cells were treated with or without ApN, while being challenged with TNFα/IFNγ. mRNA levels were normalized to TATA box-binding protein. The subsequent ratios are presented as relative expression compared to control conditions [i.e., no TNFα/IFNγ and no ApN (**a**, **b**); NT siRNA without ApN (**c**, **d**)]. **e** Utrophin A mRNAs in human myotubes, which were treated with or without ApN, while being challenged with TNFα and IFNγ. mRNA levels were normalized as above. In this last graph, each patient is represented as being its own control (statistical analysis was performed on raw paired data). Data are means ± SD; *n* = 5–6 different subjects. **p < 0.01; ***p < 0.001 vs. no TNFα/IFNγ; ###*p* < 0.001 vs. TNFα/IFNγ without ApN; §§§*p* < 0.001 vs. all other conditions

Finally, we tested the direct effects of ApN in primary cultures of human myotubes. Because TNFα expression was markedly elevated in muscles of mdx mice, and to mimic the inflammatory microenvironment of DMD, we challenged the myotubes with an inflammatory stimulus (TNFα/IFNγ) (Fig. 7). TNFα combined with IFNγ induced its own gene expression and that of another inflammatory cytokine, IL-6. ApN treatment abrogated these inductions (Fig. 7a, b).

**Fig. 8:**
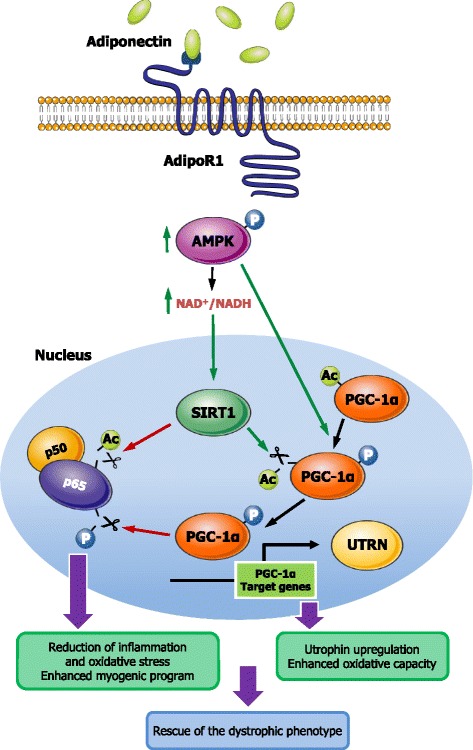
Proposed model for the protective effects of adiponectin on dystrophic muscle. Signal transduction mediating ApN protection on dystrophic muscle: binding of ApN to AdipoR1 activates the AMPK/SIRT1/PGC-1α pathway. Briefly, ApN leads to AMPK phosphorylation/activation. P-AMPK in turn phosphorylates PGC-1α and indirectly increases the expression of SIRT1 (through rising NAD+/NADH ratio). SIRT1 in turn deacetylates and fully activates PGC-1α. Next, PGC-1α represses NF-κB activity by dephosphorylation of the p65 subunit [38], while SIRT1 represses it by deacetylation [39]. This results in decreased muscle inflammation/oxidative stress and improved myogenic program as well as enhanced utrophin expression and oxidative capacity, both processes helping rescue the dystrophic phenotype. *Green arrow*, stimulation; *red arrow*, inhibition

Consistent with this, ApN reduced the rise of TNFα mRNAs in response to this pro-inflammatory cocktail or increased the mRNA levels of IκB (an inhibitor of NF-κB) in the presence of non-targeting (NT) siRNAs [compare the first two histograms (black vs. white column) of each panel; (Fig. 7c, d)]. These anti-inflammatory effects of ApN were abolished by siRNA silencing of genes encoding for either AdipoR1, SIRT1, or PGC-1α (Fig. 7c, d). Hence, each of these components appears to be necessary for the anti-inflammatory action of ApN (Fig. 8).

In this inflammatory setting, ApN treatment also slightly upregulated utrophin A mRNA in human myotubes with intact dystrophin (Fig. 7e).

## Discussion

A crucial finding of this study is that we evidenced a decrease of ApN in mdx mice. Another novel observation is that ApN correction yields striking and long-lasting attenuation of muscle inflammation and injury and improves muscle function.

We unambiguously showed that plasma ApN is decreased in mdx mice, in line with recent serum proteome data, which were however qualitative and obtained in a very limited number (*n* = 3) of mdx mice [33]. Such a decrease is relevant as it may justify therapeutic correction. We further demonstrated that it may be ascribed to reduced ApN production by fat tissue, the main contributor to systemic levels. This reduced production may in turn result from the systemic inflammatory environment in DMD [34]. TNFα indeed repressed ApN expression in adipocytes [35]. By contrast, circulating ApN was almost normalized in mdx-ApN mice due to homotopic overexpression of ApN.

As a result, mdx-ApN mice exhibited marked reduction of muscular inflammation as shown by decreased expression of pro-inflammatory cytokines and lower infiltration by both T-lymphocytes and macrophages. As in other tissues [36], ApN could also promote in muscle macrophage polarization toward an anti-inflammatory phenotype as suggested by decreased expression of CD68, a M1 marker and increased expression of IL-10, which activates the anti-inflammatory and tissue-healing M2 phenotype. Markers of oxidative stress were also reduced in mdx-ApN mice. All these data are in agreement with our previous study carried out in ApN-KO mice, which exhibited a contrario higher signs of muscular inflammation and oxidative stress, which were corrected by muscular electrotransfer of the ApN gene [6]. Herein, we further show that the presence of ApN is crucial to counterbalance inflammation and oxidative stress in skeletal muscle even when this inflammation is extremely severe and long-lasting such as in dystrophic muscle.

Besides its anti-inflammatory properties, ApN also positively affects the skeletal myogenic program. ApN has been reported to activate muscle satellite cells, which are major participants in muscle regeneration [37] and to induce differentiation and fusion of C2C12 myoblasts [26]. Herein, the skeletal muscle of mdx-ApN mice displayed partial or complete restoration of MyoD and Myf5, two positive regulatory factors of the proliferative stage [27, 28] as well as of Mrf4, a regulator of the early differentiation phase [25]. Myogenin, another marker of this early differentiation phase, was even upregulated. Taken together, these data indicate a more efficient skeletal myogenic program in mdx-ApN mice.

Both anti-inflammatory and myogenic properties may contribute to ApN protection against muscle injury. Upregulation of utrophin may also be a crucial contributor. Thus, ApN overexpression strikingly reduced both plasma CK and LDH levels and fiber membrane damage in all dystrophic muscles, as shown by in vivo staining with EBD. Accordingly, mdx-ApN mice improved global muscular force and exercise endurance in vivo. Conversely, ApN-KO mice exhibited lower peak tetanic forces in muscles stimulated in situ, while mice with muscle-specific disruption of AdipoR1 showed reduced treadmill exercise endurance [8, 38].

Although the anti-diabetic and metabolic effects of ApN are well described in skeletal muscle, the mechanisms underlying its anti-inflammatory/protective properties are only poorly studied. These molecular mechanisms were therefore investigated ex vivo and in vitro. As we did not find overt signs of apoptosis in our 8-week-old mdx mice (data not shown), we did not explore the sphingolipid ceramide pathway [39] but rather the AMPK axis. Phosphorylated AMPK and SIRT1 expression were reduced in skeletal muscle of mdx mice, while ApN partially or completely restored these levels. The activity of PGC-1α is enhanced by AMPK via phosphorylation and by SIRT1 via deacetylation [8]. Here, ApN stimulated PGC-1α deacetylation in mdx mice. Stimulation of the AMPK-SIRT1-PGC-1α may protect against muscle dystrophy by activating several biological processes [30]. First is upregulation of utrophin A together with the induction of a muscle fiber shift toward a more oxidative phenotype. Recent work has shown that pharmacological interventions (AICAR,…) that induce a more oxidative phenotype conferred morphological and functional improvements in mdx mice [31]. However, the induction of utrophin A appears to be a prerequisite to observe these benefits, which were detectable only in mdx mice, but not in double-KO mice (mdx mice which were also deficient in utrophin). Several studies have reported that utrophin can restore sarcolemmal integrity and serve as a surrogate to dystrophin in dystrophic muscle [32]. In this paper, utrophin appears to be a new target of ApN. Second is repression of NF-κB activity. PGC-1α has been reported to repress the phosphorylation of NF-κB family member p65, thereby inhibiting its transcriptional activity and subsequent pro-inflammatory cytokine production in C2C12 cells [40]. SIRT1 could also suppress NF-κB signaling via deacetylation of the p65 subunit [41]. Repressing NF-κB activity is relevant in the context of dystrophinopathy: on one hand, NF-κB signaling is persistently elevated in DMD mouse models and patients [11], and suppresses the myogenic program [27, 28]; on the other hand, inhibiting this pathway has been reported to exert protective effects in mouse models of muscle injury [11, 42]. We also originally found that AdipoR1 as well as SIRT1 and PGC-1α were crucial components of the anti-inflammatory action of ApN in human myotubes, a finding that seems to be verified in human dystrophic muscle cells (S. Lecompte, M. Abou-Samra, R. Boursereau, L. Noel, S.M. Brichard: Anti-inflammatory potential of Adiponectin in human dystrohic myotubes, in preparation). Third, additional biological processes not studied herein could also participate (like ApN- [5] and AMPK-mediated autophagic removal of dysfunctional proteins [29]). Taken together, our data suggest that ApN exerts its protective effects on skeletal muscle, by binding to AdipoR1 and activating the AMPK-SIRT1-PGC-1α pathway, thereby leading to NF-κB repression and utrophin upregulation (Fig. 8). Compared to other pharmacological agents targeting this signaling cascade in mdx mice (for review, see [29]), the improvement brought about by ApN, at physiological concentrations, is impressive and long-lasting.

The only medications so far shown to be effective in delaying the progression of DMD are glucocorticoids [43, 44]. However, adverse effects of glucocorticoids must be considered: growth retardation and weight gain, bone demineralization and fracture risk, cataracts, as well as behavioral disturbance in DMD boys. Other side effects involve glucose intolerance, hypertension, gastrointestinal problems, and Cushingoid appearance [43, 45]. Conversely, long-term treatment with ApN, which has been tested so far only in mouse, was not accompanied by major side effects. Transgenic mice overexpressing human adiponectin even showed increased longevity, which was ascribed to attenuation of oxidative DNA damage [46]. ApN may offer several other advantages over glucocorticoids. Unlike glucocorticoids, ApN increases insulin sensitivity [4], does not induce muscle atrophy but rather reduces proteolysis [47], upregulates utrophin (our own data) and enhances the myogenic program, prevents obesity [12, 46], and protects against hypertension [48].

## Conclusions

Adiponectin proves to be an extremely powerful hormone that protects muscle against inflammation, oxidative stress, and injury, even when the abnormalities are extremely severe and long-lasting such as in dystrophic muscle. This raises new therapeutic perspectives not only in myopathy but also in several other diseases, in which excessive inflammation plays a triggering or worsening pathogenic role.

## Additional files

Additional file 1: Figure S1. Effects of adiponectin on markers of oxidative stress and inflammation in muscles of 1-year-old mdx mice. A pdf file containing a supplementary figure. Additional file 2: Movie S2. Effects of adiponectin on mdx mice subjected to downhill treadmill running. A short mp4 movie showing the effect of adiponectin on mdx mice endurance.

## Abbreviations

Ac-Lys: acetyl lysine
AdipoR1/R2: adiponectin receptors 1 and 2
AMPK: AMP-activated protein kinase
ApN: adiponectin
ApN mice: mice overexpressing adiponectin
ApN-KO mice: adiponectin knockout mice
BB: biceps brachii
CD3: cluster of differentiation 3
CD68: cluster of differentiation 68
CK: creatine kinase
DAB: 3,3′-diaminobenzidine staining
DMD: Duchenne muscular dystrophy
EBD: Evans blue dye
EDL: extensor digitorium longus
G: gastrocnemius
HES: hematoxylin-erythrosin-safran
HNE: 4-hydroxy-2-nonenal
IFNγ: interferon gamma
IL-10: interleukin 10
IL-1β: interleukin-1β
LDH: lactate dehydrogenase
Mdx mice: a mouse model of DMD
Mdx-ApN mice: Mdx mice that overexpress adiponectin
Myf5: myogenic factor 5
Myh1/3/6/7: myosin heavy chain 1, 3 (eMyHC), 6 (Mrf4), and 7
MyoD: myogenic differentiation
MyoG: myogenin
NF-κB: nuclear factor kappa B
P-AMPK: phosphorylated AMPK
PGC-1α: peroxisome proliferator-activated receptor-γ coactivator-1α
P-p65: phosphorylated p65 subunit of NF-κB
PPARα: peroxisome proliferator-activated receptor alpha
PRDX3/5: peroxiredoxin 3 and 5
S: soleus
SIRT1: sirtuin 1
TA: tibialis anterior
TB: triceps brachii
TBP: TATA box-binding protein
TNFα: tumor necrosis factor alpha
UTRN: utrophin
WT mice: wild-type mice

## References

[1] Ouchi N, Walsh K (2007). Adiponectin as an anti-inflammatory factor. Clin Chim Acta.

[2] Maury E, Brichard SM (2010). Adipokine dysregulation, adipose tissue inflammation and metabolic syndrome. Mol Cell Endocrinol.

[3] Yamauchi T, Nio Y, Maki T, Kobayashi M, Takazawa T, Iwabu M (2007). Targeted disruption of AdipoR1 and AdipoR2 causes abrogation of adiponectin binding and metabolic actions. Nat Med.

[4] Kadowaki T, Yamauchi T, Kubota N, Hara K, Ueki K, Tobe K (2006). Adiponectin and adiponectin receptors in insulin resistance, diabetes, and the metabolic syndrome. J Clin Invest.

[5] Liu Y, Palanivel R, Rai E, Park M, Gabor TV, Scheid MP (2015). Adiponectin stimulates autophagy and reduces oxidative stress to enhance insulin sensitivity during high-fat diet feeding in mice. Diabetes.

[6] Jortay J, Senou M, Delaigle A, Noel L, Funahashi T, Maeda N (2010). Local induction of adiponectin reduces lipopolysaccharide-triggered skeletal muscle damage. Endocrinology.

[7] Jortay J, Senou M, Abou-Samra M, Noel L, Robert A, Many MC (2012). Adiponectin and skeletal muscle: pathophysiological implications in metabolic stress. Am J Pathol.

[8] Iwabu M, Yamauchi T, Okada-Iwabu M, Sato K, Nakagawa T, Funata M (2010). Adiponectin and AdipoR1 regulate PGC-1alpha and mitochondria by Ca(2+) and AMPK/SIRT1. Nature.

[9] Dalkilic I, Kunkel LM (2003). Muscular dystrophies: genes to pathogenesis. Curr Opin Genet Dev.

[10] Blake DJ, Weir A, Newey SE, Davies KE (2002). Function and genetics of dystrophin and dystrophin-related proteins in muscle. Physiol Rev.

[11] Acharyya S, Villalta SA, Bakkar N, Bupha-Intr T, Janssen PM, Carathers M (2007). Interplay of IKK/NF-kappaB signaling in macrophages and myofibers promotes muscle degeneration in Duchenne muscular dystrophy. J Clin Invest.

[12] Bauche IB, El Mkadem SA, Pottier AM, Senou M, Many MC, Rezsohazy R (2007). Overexpression of adiponectin targeted to adipose tissue in transgenic mice: impaired adipocyte differentiation. Endocrinology.

[13] Leyens G, Donnay I, Knoops B (2003). Cloning of bovine peroxiredoxins-gene expression in bovine tissues and amino acid sequence comparison with rat, mouse and primate peroxiredoxins. Comp Biochem Physiol B: Biochem Mol Biol.

[14] Zanou N, Shapovalov G, Louis M, Tajeddine N, Gallo C, Van SM (2010). Role of TRPC1 channel in skeletal muscle function. Am J Physiol Cell Physiol.

[15] Mignion L, Magat J, Schakman O, Marbaix E, Gallez B, Jordan BF (2013). Hexafluorobenzene in comparison with perfluoro-15-crown-5-ether for repeated monitoring of oxygenation using 19F MRI in a mouse model. Magn Reson Med.

[16] Wooddell CI, Zhang G, Griffin JB, Hegge JO, Huss T, Wolff JA (2010). Use of Evans blue dye to compare limb muscles in exercised young and old mdx mice. Muscle Nerve.

[17] Hamer PW, McGeachie JM, Davies MJ, Grounds MD (2002). Evans blue dye as an in vivo marker of myofibre damage: optimising parameters for detecting initial myofibre membrane permeability. J Anat.

[18] Brussee V, Tardif F, Tremblay JP (1997). Muscle fibers of mdx mice are more vulnerable to exercise than those of normal mice. Neuromuscul Disord.

[19] Ge Q, Ryken L, Noel L, Maury E, Brichard SM (2011). Adipokines identified as new downstream targets for adiponectin: lessons from adiponectin-overexpressing or -deficient mice. Am J Physiol Endocrinol Metab.

[20] Woldt E, Sebti Y, Solt LA, Duhem C, Lancel S, Eeckhoute J (2013). Rev-erb-alpha modulates skeletal muscle oxidative capacity by regulating mitochondrial biogenesis and autophagy. Nat Med.

[21] Delaigle AM, Jonas JC, Bauche IB, Cornu O, Brichard SM (2004). Induction of adiponectin in skeletal muscle by inflammatory cytokines: in vivo and in vitro studies. Endocrinology.

[22] Maury E, Noel L, Detry R, Brichard SM (2009). In vitro hyperresponsiveness to tumor necrosis factor-alpha contributes to adipokine dysregulation in omental adipocytes of obese subjects. J Clin Endocrinol Metab.

[23] Porter JD, Khanna S, Kaminski HJ, Rao JS, Merriam AP, Richmonds CR (2002). A chronic inflammatory response dominates the skeletal muscle molecular signature in dystrophin-deficient mdx mice. Hum Mol Genet.

[24] Barton ER, Morris L, Musaro A, Rosenthal N, Sweeney HL (2002). Muscle-specific expression of insulin-like growth factor I counters muscle decline in mdx mice. J Cell Biol.

[25] Tidball JG, Villalta SA (2010). Regulatory interactions between muscle and the immune system during muscle regeneration. Am J Physiol Regul Integr Comp Physiol.

[26] Fiaschi T, Cirelli D, Comito G, Gelmini S, Ramponi G, Serio M (2009). Globular adiponectin induces differentiation and fusion of skeletal muscle cells. Cell Res.

[27] Langen RC, Van Der Velden JL, Schols AM, Kelders MC, Wouters EF, Janssen-Heininger YM (2004). Tumor necrosis factor-alpha inhibits myogenic differentiation through MyoD protein destabilization. FASEB J.

[28] Guttridge DC, Mayo MW, Madrid LV, Wang CY, Baldwin AS (2000). NF-kappaB-induced loss of MyoD messenger RNA: possible role in muscle decay and cachexia. Science.

[29] Ljubicic V, Burt M, Jasmin BJ (2014). The therapeutic potential of skeletal muscle plasticity in Duchenne muscular dystrophy: phenotypic modifiers as pharmacologic targets. FASEB J.

[30] Chan MC, Arany Z (2014). The many roles of PGC-1alpha in muscle—recent developments. Metabolism.

[31] Al-Rewashdy H, Ljubicic V, Lin W, Renaud JM, Jasmin BJ (2015). Utrophin A is essential in mediating the functional adaptations of mdx mouse muscle following chronic AMPK activation. Hum Mol Genet.

[32] Squire S, Raymackers JM, Vandebrouck C, Potter A, Tinsley J, Fisher R (2002). Prevention of pathology in mdx mice by expression of utrophin: analysis using an inducible transgenic expression system. Hum Mol Genet.

[33] Hathout Y, Marathi RL, Rayavarapu S, Zhang A, Brown KJ, Seol H (2014). Discovery of serum protein biomarkers in the mdx mouse model and cross-species comparison to Duchenne muscular dystrophy patients. Hum Mol Genet.

[34] Pan Y, Chen C, Shen Y, Zhu CH, Wang G, Wang XC (2008). Curcumin alleviates dystrophic muscle pathology in mdx mice. Mol Cells.

[35] Ruan H, Lodish HF (2003). Insulin resistance in adipose tissue: direct and indirect effects of tumor necrosis factor-alpha. Cytokine Growth Factor Rev.

[36] Ohashi K, Parker JL, Ouchi N, Higuchi A, Vita JA, Gokce N (2010). Adiponectin promotes macrophage polarization toward an anti-inflammatory phenotype. J Biol Chem.

[37] Fiaschi T, Giannoni E, Taddei ML, Chiarugi P (2012). Globular adiponectin activates motility and regenerative traits of muscle satellite cells. PLoS ONE.

[38] Krause MP, Liu Y, Vu V, Chan L, Xu A, Riddell MC (2008). Adiponectin is expressed by skeletal muscle fibers and influences muscle phenotype and function. Am J Physiol Cell Physiol.

[39] Holland WL, Miller RA, Wang ZV, Sun K, Barth BM, Bui HH (2011). Receptor-mediated activation of ceramidase activity initiates the pleiotropic actions of adiponectin. Nat Med.

[40] Eisele PS, Salatino S, Sobek J, Hottiger MO, Handschin C (2013). The peroxisome proliferator-activated receptor gamma coactivator 1alpha/beta (PGC-1) coactivators repress the transcriptional activity of NF-kappaB in skeletal muscle cells. J Biol Chem.

[41] Yang XD, Tajkhorshid E, Chen LF (2010). Functional interplay between acetylation and methylation of the RelA subunit of NF-kappaB. Mol Cell Biol.

[42] Siegel AL, Bledsoe C, Lavin J, Gatti F, Berge J, Millman G (2009). Treatment with inhibitors of the NF-kappaB pathway improves whole body tension development in the mdx mouse. Neuromuscul Disord.

[43] Beytia ML, Vry J, Kirschner J (2012). Drug treatment of Duchenne muscular dystrophy: available evidence and perspectives. Acta Myol.

[44] Manzur AY, Kuntzer T, Pike M, Swan A (2008). Glucocorticoid corticosteroids for Duchenne muscular dystrophy. Cochrane Database Syst Rev.

[45] Moxley RT, Pandya S, Ciafaloni E, Fox DJ, Campbell K (2010). Change in natural history of Duchenne muscular dystrophy with long-term corticosteroid treatment: implications for management. J Child Neurol.

[46] Otabe S, Yuan X, Fukutani T, Wada N, Hashinaga T, Nakayama H (2007). Overexpression of human adiponectin in transgenic mice results in suppression of fat accumulation and prevention of premature death by high-calorie diet. Am J Physiol Endocrinol Metab.

[47] Zhou Q, Du J, Hu Z, Walsh K, Wang XH (2007). Evidence for adipose-muscle cross talk: opposing regulation of muscle proteolysis by adiponectin and fatty acids. Endocrinology.

[48] Wang ZV, Scherer PE (2008). Adiponectin, cardiovascular function, and hypertension. Hypertension.

