# Supplementary material for: Involvement of adiponectin in the pathogenesis of dystrophinopathy
Source: Skelet Muscle. 2015 Aug 7;5:25. doi: 10.1186/s13395-015-0051-9 (PMC4528853; doi:10.1186/s13395-015-0051-9)
Supplement: Additional file 1: Figure S1. — Effects of adiponectin on markers of oxidative stress and inflammation in muscles of 1-year-old mdx mice. A pdf file containing a supplementary figure. [file 13395_2015_51_MOESM1_ESM.pdf]

SUPPLEMENTARY INFORMATION

Additional file 1: Figure S1

Fig.S1.

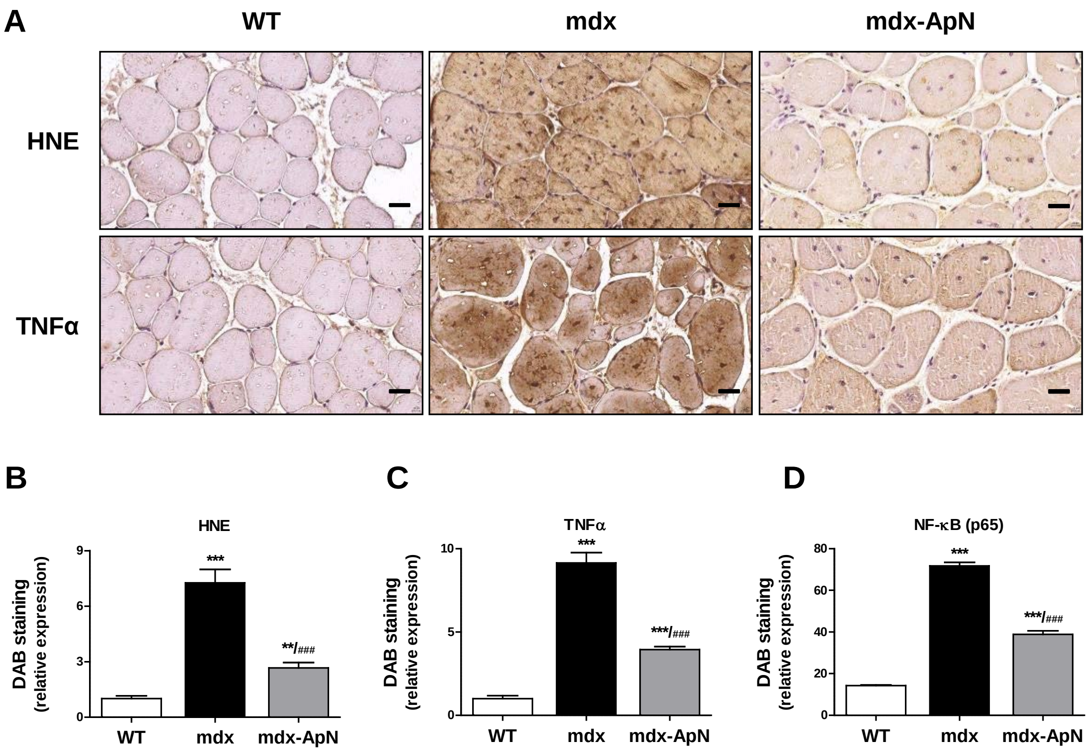

Fig. S1. Effects of adiponectin on markers of oxidative stress and inflammation in muscles of 1 yr-old mdx mice. (A) *Tibialis anterior* muscles were sampled from 1 yr-old WT mice, mdx mice or mdx mice overexpressing adiponectin (mdx-ApN). Immunodetection was performed with specific antibodies directed against one pro-inflammatory cytokine (TNFα), and one oxidative stress marker (HNE). Scale bar = 25 µm. **(B)** HNE and **(C)** TNFα immunolabeling, the percentage of DAB deposit areas was calculated within the muscle fibers. **(D)** Quantification of p65 immunolabeling in myofiber nuclei (expressed as percent of total nuclei). Data are means ± SD for 4 mice and are presented relative to DAB ratio in WT mice.

** $p < 0.01$, *** $p < 0.001$ *vs.* WT. ### $p < 0.001$ *vs.* mdx.

Additional file 2: Movie S2

Movie S2. Effects of adiponectin on mdx mice subjected to downhill treadmill running. Mice (*n*=6/group) were subjected to an eccentric exercise consisting of a 10 min downhill running on a treadmill with an inclination of 15°, at a speed of 10 m/min, and then repeated daily for 3 days. When mice stopped exercising, a mild electrical current was discharged by an electric grid (indicated by a star) to keep them running on the treadmill. A representative video shows the difference of global muscular force between a regular mdx mouse (on the right) and a mdx-ApN mouse (on left) on the 3rd day. Mdx mice struggled during the last session of the exercise and remained most of the time on -or nearby- the electric grid, while most mdx-ApN mice completed the exercise.
